# Temporal Trends in Dietary Macronutrient Intakes among Adults in Rural China from 1991 to 2011: Findings from the CHNS

**DOI:** 10.3390/nu9030227

**Published:** 2017-03-05

**Authors:** Chang Su, Jian Zhao, Yang Wu, Huijun Wang, Zhihong Wang, Yun Wang, Bing Zhang

**Affiliations:** 1 National Institute for Nutrition and Health, Chinese Center for Disease Control and Prevention, Beijing 100050, China; suchang@chinacdc.cn (C.S.); zhaojian131023@163.com (J.Z.); wanghj128@gmail.com (H.W.); wangzh@chinacdc.cn (Z.W.); wangyunn@aliyun.com (Y.W.); 2 Bloomberg School of Public Health, Johns Hopkins University, Baltimore, MD 21205, USA; wuyangathy@gmail.com

**Keywords:** rural population, nutrition transition, macronutrients, China

## Abstract

Few studies have examined nutrition transitions among the rural population of China, even though half of the Chinese population (about 700 million) is living in rural China. To fill this research gap, we examined temporal trends in dietary macronutrient intakes in members of the Chinese rural population aged 18–60 years. The analysis used data from consecutive three-day dietary recalls, collected from the China Health and Nutrition Surveys (CHNS). Mixed-effect models were constructed to obtain adjusted means and to examine temporal trends after adjusting for intra-class correlation within clusters and covariates, including age, sex, geographical region, urbanicity, and income. From 1991 to 2011, a downward trend in daily energy, protein, and carbohydrate intakes was seen in all categories, with a significant reduction among all rural people (*p* < 0.0001). In contrast, a significant increment in daily fat intake, the proportion of energy from fat, and the proportion of rural people consuming a diet with more than 30% of energy from fat, were observed in the present study (*p* < 0.0001). These results suggest that adults in rural China have been undergoing a rapid nutrition transition towards a high-fat diet. Therefore, more emphasis should be placed on the quality of fat and maintaining a balanced diet during the process of nutritional education.

## 1. Introduction

In recent years, dietary patterns have shifted towards a diet that is high in saturated fats, cholesterol, and sugar, but low in unsaturated fats and fiber, and these have been regarded as an important contributor to the increasing prevalence of chronic diseases, including obesity, cardiovascular diseases, and cancers [1,2,3,4,5]. A growing number of studies have shown that the shift in dietary patterns has contributed to a large disease burden [3,6]. China has been undergoing a dramatic transition in disease and dietary patterns, accompanied by rapid economic growth, lifestyle changes, and population aging [7,8].

Most previous studies have focused on dietary pattern shifts in the Chinese urban population of all age groups [4,5,9,10,11,12,13], but have ignored the rural residents. In China, a higher proportion of rural residents are below the average poverty levels, and are also more likely to fall victim to over-nutrition or under-nutrition than urban residents [9]. Mortality in the rural population is also substantially higher than that in the urban population [9]. In addition, China’s population is ageing rapidly. There are currently 618 million rural residents, making up almost half of China’s total population. Therefore, more attention should be paid to the nutrition transition in rural communities. Although there have been some reports on the lifestyles and status of chronic diseases in rural people in China, few studies have focused on the trends in nutrient intakes. Moreover, evaluations on food consumption trends could help social agencies to gain some insight into the implementation and monitoring of health and nutrition policies, as well as evaluations on how public health information is perceived and actualized by the population [14]. The present study aimed to examine the secular trends in dietary energy and macronutrient intake among rural Chinese groups between 1991 and 2011, using data from the China Health and Nutrition Survey (CHNS).

## 2. Materials and Methods

### 2.1. Study Population

The data used in the present study were drawn from the China Health and Nutrition (CHNS), which is a longitudinal household survey with ongoing data collection across 239 communities within nine out of all 31 provinces in mainland China. The surveys began in 1989 and have been followed up every two to four years. So far, there have been a total of nine waves of surveys, conducted in 1989, 1991, 1993, 1997, 2000, 2004, 2006, 2009, and 2011. As the only large-scale, longitudinal study of this kind in China, the CHNS aims to collect representative data on rural and urban areas in China, with substantial variations in sociological, economic, and demographic transformations, and to assess their effects on the health and nutritional status of the Chinese population. Participants in the CHNS were selected based on a multistage cluster random sampling scheme in each province, and the sampling scheme is reported in detail elsewhere [10]. Essentially, two cities (a provincial capital and a lower income city) and four counties (stratified by income: one low-, two middle-, and one high-income county, defined on the basis of per-capita income) were randomly selected from each province. Within the cities, two urban and two suburban neighborhoods were chosen; within the counties, the township capital and three villages were selected. The neighborhoods and villages were defined as communities in this study. Twenty households were then randomly selected from each community and all individuals in each household were interviewed.

Our analysis used eight rounds of survey data between 1991 and 2011, as the 1989 survey only consisted of young adults aged 20–45 years old. Of all the rural adults (aged 18–60) with complete data on socioeconomic status, demographics, and three-day, 24 h dietary recalls in a survey year, we excluded pregnant or lactating women, and those which had implausible energy intakes (<800 kcal/day or >6000 kcal for men and <600 kcal or >4000 kcal for women) [15]. The current analysis therefore consisted of 12,523 participants (6046 males and 6477 females) clustered in 239 communities, resulting in 41,739 total responses across the eight survey years. The Survey protocols, instruments, and the process for obtaining informed consent, were approved by the National Institute for Nutrition and Health, the Chinese Center for Disease Control and Prevention, and the Institutional Review Committees of the University of North Carolina at Chapel Hill (No. 201524-1). Written informed consent was obtained from all subjects.

### 2.2. Dietary Data Collection

Dietary assessment was based on a combination of data collected from three consecutive 24 h recall surveys, which included two weekdays and one weekend at the individual level, as well as a food inventory taken at the household level over the same three-day period. Household food consumption was determined by examining changes in the inventory from the beginning to the end of the survey, in combination with a weighing and measuring technique. All foods and condiments (including edible oil), no matter if they were purchased from markets, picked from gardens, or were food waste, were weighed and recorded. Individual dietary intake data were collected by asking each household member to report all of the food which they had consumed at home and away from home on a three-day, 24 h recall basis. Using picture aids and food models, trained field interviewers recoded the types, amounts, types of meal, and places of consumption for each food consumed during the previous day. The amount of food in each dish was estimated from the household inventory and the proportion of each dish which was consumed was reported by each person that was interviewed [16]. The quality of the data collection was controlled in two ways. Firstly, all field workers had previously taken a high-quality training course for at least three days before the collection of dietary data [16,17,18]. Following the data collection, well-trained interviewers compared an individual’s average daily dietary intake calculated from the household inventory, with the individual’s dietary intake based on the 24 h recall surveys. Where significant differences were found, the interviewers revisited the households and individuals in question, and further inquired about the individual food consumption.

The Chinese Food Composition Table (1991) was utilized to calculate each individual’s daily intake of select nutrients for each food item in the dietary data, from 1991 to 2000. An updated version of Food Composition Table (2002 and 2004) was used for the 2000, 2004, and 2006 surveys, and the latest version (2009) was used for the 2009 and 2011 surveys [19].

### 2.3. Other Variables

Per capita annual household income was calculated using the following formula: annual household income/household size. The per capita annual household income in each survey was inflated to values in 2011 by adjusting for the consumer price index, and was then recoded into tertiles (low-, medium-, and high-level). Resident locations were divided into three areas, based on geographic location: North (Heilongjiang and Liaoning), Central (Shandong, Jiangsu, and Henan), and South (Hubei, Hunan, Guangxi, and Guizhou). Urbanicity is defined by multi-component scales composed of 12 domains with 10 points representing each domain, including population density, economic activity, traditional markets, modern markets, transportation infrastructure, sanitation, communications, housing, education, diversity, health infrastructure, and social services, which has been explained in previous studies [20,21,22]. A higher urbanicity score indicated that the community has a higher degree of urbanicity. The scales developed for CHNS have a high validity, reliability, and temporal stability [21,23]. The final urbanicity index was calculated from the total scores of the 12 domains, and these indexes were then categorized into tertiles (low-, medium-, and high-level), to reflect the degree of urbanicity for each community over the time period of the present study.

### 2.4. Statistical Analysis

The values were reported as means and standard errors for continuous variables, or as proportions of the total for categorical variables. Data were subdivided according to different demographic characteristics. Adjusted means and standard errors were used to describe the distributions of continuous variables, after adjusting for complex sampling and covariates, including age, sex, region, per capita annual household income, and urbanicity. Age was adjusted as a continuous variable, and household income and urbanicity were adjusted as categorical variables. Linear mixed-effect models were produced to calculate the adjusted mean intakes of total energy, carbohydrate, protein, fat, and macronutrient-energy percentage, and to explore the temporal trends after adjusting for intra-class correlation within clusters and covariates, including age, sex, residence, income levels, and urbanicity levels. All statistical analyses were conducted using SAS 9.1 software (SAS Institute, Cary, NC, USA).

## 3. Results

Table 1 presents the characteristics of the rural adult population aged 18–60 years in the CHNS, by survey year. The sample size was 4926 in 1991, 5212 in 1993, 5441 in 1997, 6036 in 2000, 5318 in 2004, 5112 in 2006, 5134 in 2009, and 4560 in 2011. The mean age ranged from 36.2 to 43.7 years, and there were statistically significant differences across the survey periods (*p* < 0.01). There were no significant differences in the distribution of sex across the survey years. However, we observed significant temporal trends in family income and urbanicity across the survey years (*p* < 0.01), which indicated that rapid economic growth and dramatic urbanicity have occurred in the past 20 years in China.

As shown in Table 2, energy intakes among the Chinese rural population steadily declined over time across all age (18–39 and 40–60 years), sex, region, urbanicity, and income groups (*p* < 0.0001). The average daily energy intake decreased from 2512.7 kcal in 1991, to 2192.0 kcal in 2011. It is worth noting that the decline in energy intakes of rural adults in the younger age group (355.9 kcal/day), was larger than that in the older group (282.8 kcal/day). Moreover, the largest decline in energy intake, of 391.8 kcal/day, was found in low-income groups. This decline was larger than that in the medium- (306.2 kcal/day) or high-income groups (286.6 kcal/day).

Likewise, as seen in Table 3, the daily carbohydrate intake steadily dropped across the survey years in each age group, sex, region, urbanicity, and different income group (*p* < 0.0001). The daily carbohydrate intake dropped from 394.8 g in 1991, to 319.4 g in 2011. The decline in carbohydrate intake in rural women was higher than that in rural men. Furthermore, the decline in carbohydrate intake in the rural population from the low-income group was higher than that in medium- and high-income groups.

**Figure 1:**
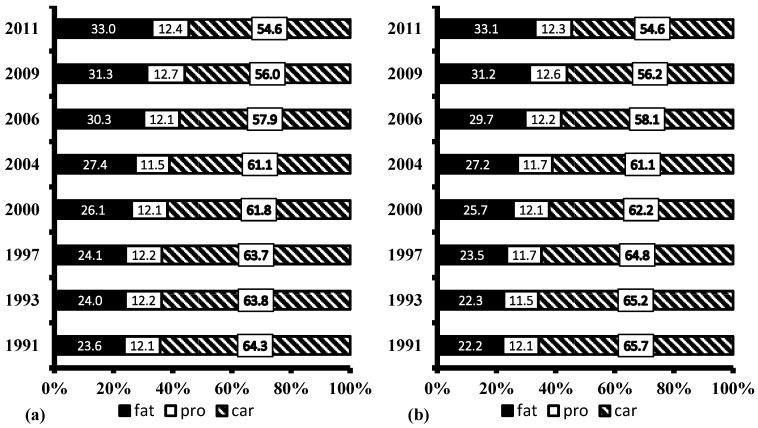
Percentages of energy from protein, carbohydrate and fat in Chinese rural adults from 1991 to 2011 by gender: (**a**) male; (**b**) female. Proportions were adjusted for age, sex, region, income, urbanicity. There was as significant trend in the proportions of energy from fat and carbohydrate across the survey years (*p* < 0.0001; test for trend).

Table 4 shows that the daily fat intake in the Chinese rural population increased from 65.8 to 76.9 g (*p* < 0.0001), from 1991 to 2011. Meanwhile, the energy intake of rural residents substantially decreased. As a result, the proportion of energy from fat increased, while the proportion of energy from carbohydrate decreased, from 1991 to 2011, in both males and females (Figure 1). The proportion of the rural population consuming a diet with more than 30% of energy from fat increased from 24.6% in 1991, to 58.3% in 2011 (Table 5). Furthermore, the increase in the proportion of the rural population who consumed more than 30% of energy from fat was greater in the low-income group than in high-income group, while the increase was larger in the high-urbanicity group than in the low-urbanicity group.

Table 6 presents the daily protein intake among the Chinese rural population from 1991 to 2011, which steadily declined from 75.3 g to 64.4 g during this period (*p* < 0.0001). We observed that the daily protein intake steadily dropped across the survey years in each age group, sex, region, urbanicity, and different income groups (*p* < 0.0001).

## 4. Discussion

Previous studies have evaluated the nutritional status and nutrition transition in different countries [24,25,26,27,28], but no previous studies have assessed the dietary energy and macronutrient intakes in Chinese rural people across three levels of urbanicity, in three regions of China. The present study shows that dietary energy intake has dramatically decreased between 1991 and 2011, while the dominant dietary pattern has shifted from one high in carbohydrate, to one high in fat. The proportion of rural people consuming a high fat diet more than tripled from 1991 to 2011. Findings from the present study indicate that Chinese rural people have been undergoing a rapid nutrition transition in the past two decades, supported by economic growth and rapid modernisation. It is especially noted that the largest transitions in dietary patterns took place in rural people from low-income households. Our results suggest that the disease burdens brought about by the nutrition transition may shift toward low-income rural people in China in the future.

Our findings indicated that the total energy intake in Chinese rural populations significantly declined during the past two decades. The observed trend is consistent with that observed in an urban population from 2394.6 kcal in 1992, to 2053.0 kcal in 2012 [29,30]. However, the decline in energy intake in Chinese urban people during the same period was much larger than that in rural people between the 1990s and 2010s. These findings are also in agreement with a previous study, which showed a steady decrease in the total energy intake for Indian rural women, from 2014.0 kcal in 1997 to 1780.0 kcal in 2011, in parallel with economic development [31]. Given the rapid increase in the prevalence of overweight and obese Chinese people, a potential explanation for this decline in the total energy intake is a decrease in the total energy expenditure. In our previous study, it was illustrated that both occupational and domestic activity decreased, and sedentary hours increased in the Chinese population during the past two decades [32]. There are also other reasons for such a decline, such as an increased underestimation of dietary intake [33], and it would be important to distinguish the outcomes of these factors in future research.

A notable change in the total fat intake and the proportion of energy from fat has occurred in Chinese rural adults during the last two decades. In both genders, the total fat intake increased from 1991 to 2011, by 18.4% in men and by 15.6% in women. Similar trends have been found in the urban population enrolled in the China National Nutrition and Health Survey (CHNNS) between 1992 and 2012 [29,30]. Most previous studies have reported a positive link between the increased total fat intake in adults worldwide, and economic growth and nutrition transitions in the past several decades [24,25,26]. Contrary to our results, the total fat intake in Indian rural women decreased from a very low fat consumption of 25.2 g in 1997, to 25.1 g in 2011 [31]. It is worth noting that an increase in the consumption of total fat was also observed in South Korea [28], but South Koreans maintained a low- fat, traditional diet, because of the nationwide campaign advocating healthy eating habits. Hence, if reasonable measures are taken, a traditional diet can be maintained, despite rapid economic growth.

As a main source of dietary energy, carbohydrate plays a critical role in the Chinese traditional diet. A continuous decrease in carbohydrate intake was observed, which is in agreement with a previous study that confirmed a decline of carbohydrate consumption in Chinese urban adults from 348.7 g in 1992, to 261.1 g in 2012 [29,30]. The decrease in carbohydrate can partly be explained by the drop in energy expenditure, as Chinese lifestyles have become more sedentary [32]. The drop in the carbohydrate intake in Chinese rural adults was similar to that found in adults in some developing Asian countries, but was inconsistent with adults in Morocco and Chile [24,25], where the consumption of carbohydrate increased, resulting in an increase in the total energy intake over the past four decades.

A dramatic decline was observed regarding the consumption of total protein in rural adults’ diets, a result also recorded elsewhere [31]. Our results are supported by the official data [29], which showed a decrease in protein intake of 3% in 2002 relative to 1982, and also by a previous study, which showed a slow decrease in protein intake in women between 1997 and 2011 [31]. The reasons for the decline in protein intake are difficult to identify and can only be speculated. For instance, a potential explanation may be due to significantly reduced plant food. Future efforts are needed to explore the other impossible reasons.

The Chinese Dietary Guideline (CDG) of 2016 suggested that, for Chinese adults, the total energy derived from carbohydrate, fat, and protein, was 55%–65%, below 30%, and 10%–15%, respectively [34]. The present study indicates that the total energy intakes derived from carbohydrate, protein, and fat in males (54.6%, 12.4%, and 33.0%, respectively) were very similar to those (54.6%, 12.3%, and 33.1%, respectively) in females in 2011. The proportion of total energy from protein and carbohydrate is in line with the CDG recommendation, but the proportion of total energy from fat exceeded the recommendation. Moreover, the proportion of rural adults consuming a diet with more than 30% of total energy from fat more than tripled from 1991 to 2011 (nearly 60%). Excessive fat intake may lead to the development of NCD and chronic conditions such as hypertension [35], overweight and obesity [36,37], and type 2 diabetes [38]. Accordingly, the Chinese government need to take immediate actions to implement effective interventions which promote a healthy diet, considering a rapid acceleration of nutrition and epidemiological transition with an increased burden of NCDs [39]. These measures are especially needed in China’s southern rural regions, where approximately two-thirds of the Chinese rural people had relative fat intakes higher than those recommended by CDG.

Relative to many parts of the developing world, as the biggest developing country, a double burden of undernutrition and overnutrition have been observed in the Chinese population [40]. About 50% of China’s population resides in rural China. Experiencing a faster change of dietary pattern, the nutrition status in the rural population is gradually shifting from undernutrtion to overnutrition. The excess of the total energy intake and macronutrients is known to have a negative impact on a population’s health and can lead to an increased risk of NCD [36]. Apart from the influence of rapid nutrition transition, the rural populations are also at a higher risk of NCDs because of low health literacy, compared to the urban population [41,42]. Currently, some food programs have been pursued by the Chinese government, which target the rural population and aim to improve their dietary quality and promote the development of better eating habits. These programs could have great effects on controlling and preventing the rapid increase of NCDs.

The present study has many strengths, including its large sample size with a wide age range, which was carried out by staff trained in the study’s methodology and the simultaneous standardization of different parameters, by the same scientists. The use of the individual, consecutive three-day recall method could improve the accuracy of dietary recalls and hence, the analysis and results [43]. Moreover, mixed-effect modeling could reduce bias and increase the accuracy of estimates.

Several limitations warrant cautious interpretations of our findings. First, dietary data were collected using three consecutive 24-h dietary recalls, which might have relatively limited corrections for within-subject variations, compared to non-consecutive 24-h recalls. However, the average intake over three days can offer a relatively valid estimate of nutrient intakes, as shown in an earlier study using the CHNS [44]. Secondly, individuals may report their food consumption inaccurately because of various reasons such as memory, knowledge, and the interview situation [31]. Furthermore, it is possible that the obese participants might have under-reported their true habitual food intake, compared with non-obese participants [45]. In addition, the CHNS does not present national data and the vast, western areas of China were not included in the present study.

## 5. Conclusions

The potential problems of the rapid nutrition transition in the rural Chinese population, especially in those from low-income or low-urbanicity groups, have important policy implications. Public health policies should pay special attention to dietary intakes, especially the quality of fat, to ensure a more balanced diet for adults in rural China.

## Figures and Tables

**Table 1:** General characteristics of participants aged 18–60 years.

General Characteristics	Survey Year ^1^							
	1991	1993	1997	2000	2004	2006	2009	2011
*n*	4926	5212	5441	6036	5318	5112	5134	4560
Age (year) ^2^	36.2 ± 0.2 ^2^	36.4 ± 0.2	37.8 ± 0.2	39.1 ± 0.1	41.7 ± 0.2	42.6 ± 0.2	42.7 ± 0.2	43.7 ± 0.2
18–39 (%)	61.4	59.7	53.9	50.4	41.7	38.3	37.6	32.6
40–60 (%)	38.6	40.3	46.1	49.6	58.3	61.7	62.4	67.4
Male (%)	47.7	48.5	50.6	50.0	48.9	48.5	48.5	47.5
Income (CNY) ^3^								
Low	4.0 ± 0.1	4.1 ± 0.1	5.1 ± 0.1	5.3 ± 0.1	6.0 ± 0.1	6.4 ± 0.1	10.3 ± 0.1	12.1 ± 0.2
Medium	9.6 ± 0.1	10.3 ± 0.1	12.8 ± 0.1	15.1 ± 0.1	16.8 ± 0.1	18.9 ± 0.1	28.2 ± 0.1	34.4 ± 0.2
High	22.1 ± 0.3	25.9 ± 0.3	30.8 ± 0.4	38.4 ± 0.6	46.5 ± 0.6	57.5 ± 1.2	78.8 ± 1.3	92.5 ± 1.6
Urbanicity (score) ^2^								
Low	25.5 ± 0.1	26.6 ± 0.1	28.8 ± 0.1	35.2 ± 0.1	36.9 ± 0.1	38.4 ± 0.1	42.4 ± 0.1	44.4 ± 0.1
Medium	36.9 ± 0.1	39.7 ± 0.1	42.1 ± 0.1	47.3 ± 0.1	49.3 ± 0.1	51.9 ± 0.1	55.3 ± 0.1	58.1 ± 0.1
High	52.8 ± 0.2	55.9 ± 0.2	61.5 ± 0.2	67.5 ± 0.2	73.0 ± 0.3	75.6 ± 0.2	77.8 ± 0.3	80.1 ± 0.2

^1^ Significant trend in the subgroup of age, income, and urbanicity across the survey years (*p* < 0.01; test for trend). Percentage of males did not significantly change over time (*p*-trend > 0.05). ^2^ Mean ± SE (all such values). ^3^ Adjusted to Chinese yuan value in 2011 (per 1000 yuan).

**Table 2:** Daily energy intake (Kcal), by age, sex, geographical region, income, and urbanicity, in Chinese rural adults from 1991 to 2011.

General Characteristics	Survey Year ^1^								*p*_trend_^2^
	1991	1993	1997	2000	2004	2006	2009	2011	
All	2512.7 ± 43.7	2411.7 ± 43.9	2398.7 ± 43.6	2393.5 ± 43.8	2348.8 ± 43.7	2344.2 ± 43.8	2273.0 ± 43.8	2192.0 ± 44.0	*p* < 0.0001
Age (year)									
18–39	2553.0 ± 37.0	2428.1 ± 37.1	2413.1 ± 36.9	2395.0 ± 37.0	2354.0 ± 38.0	2352.2 ± 37.6	2285.3 ± 38.2	2197.1 ± 39.4	*p* < 0.0001
40–60	2472.9 ± 66.2	2402.1 ± 65.9	2391.9 ± 65.9	2388.3 ± 66.0	2345.1 ± 66.8	2340.5 ± 66.0	2244.2 ± 66.6	2190.1 ± 66.1	*p* < 0.0001
Sex									
Male	2740.2 ± 46.6	2622.3 ± 46.2	2602.3 ± 46.2	2557.2 ± 46.1	2535.3 ± 46.6	2477.3 ± 46.4	2475.7 ± 46.4	2422.9 ± 46.9	*p* < 0.0001
Female	2297.8 ± 45.5	2296.4 ± 45.7	2221.7 ± 45.6	2209.5 ± 45.3	2181.6 ± 45.5	2155.6 ± 45.5	2086.4 ± 45.6	1979.8 ± 45.9	*p* < 0.0001
Region									
North	2507.4 ± 30.7	2380.1 ± 30.5	2374.0 ± 29.6	2263.5 ± 27.8	2209.5 ± 27.6	2205.6 ± 27.3	2153.6 ± 28.0	2077.6 ± 28.9	*p* < 0.0001
Central	2526.6 ± 81.1	2520.7 ± 81.0	2488.8 ± 81.5	2459.6 ± 81.5	2382.4 ± 81.4	2368.8 ± 81.9	2358.9 ± 81.3	2313.5 ± 81.3	*p* < 0.0001
South	2493.5 ± 86.2	2441.2 ± 86.2	2424.2 ± 86.0	2408.4 ± 86.2	2367.8 ± 86.2	2354.0 ± 86.1	2243.6 ± 86.3	2177.3 ± 86.5	*p* < 0.0001
Income									
Low	2595.4 ± 58.3	2440.4 ± 58.5	2428.4 ± 58.0	2421.9 ± 58.8	2357.4 ± 58.6	2328.7 ± 58.3	2262.1 ± 58.8	2203.6 ± 59.4	*p* < 0.0001
Medium	2541.8 ± 57.8	2447.4 ± 58.3	2433.8 ± 57.9	2401.3 ± 57.5	2366.8 ± 58.1	2353.8 ± 57.8	2299.2 ± 58.0	2235.6 ± 58.3	*p* < 0.0001
High	2451.5 ± 39.7	2381.7 ± 40.2	2379.1 ± 39.3	2374.9 ± 39.6	2336.1 ± 39.7	2331.7 ± 39.9	2276.7 ± 39.6	2164.9 ± 40.0	*p* < 0.0001
Urbanicity									
Low	2586.3 ± 81.6	2457.9 ± 81.7	2433.7 ± 58.0	2384.0 ± 81.4	2375.7 ± 81.6	2325.5 ± 81.5	2309.3 ± 81.8	2262.2 ± 82.0	*p* < 0.0001
Medium	2540.3 ± 52.9	2448.5 ± 52.6	2436.2 ± 53.1	2427.5 ± 53.3	2394.5 ± 52.9	2366.0 ± 53.1	2321.2 ± 53.2	2104.7 ± 53.6	*p* < 0.0001
High	2456.5 ± 48.1	2383.9 ± 47.9	2372.1 ± 47.5	2359.3 ± 48.0	2333.5 ± 47.7	2265.6 ± 47.8	2178.4 ± 47.9	2150.7 ± 48.3	*p* < 0.0001

^1^ Mean ± SE (all such values). ^2^ Values adjusted for age, sex, region, income, and urbanicity.

**Table 3:** Daily carbohydrate intake (g), by age, sex, geographical region, income, and urbanicity, in Chinese rural adults from 1991 to 2011.

General Characteristics	Survey Year ^1^								*p*_trend_^2^
	1991	1993	1997	2000	2004	2006	2009	2011	
All	394.8 ± 7.6	372.2 ± 7.7	363.6 ± 7.6	363.2 ± 7.6	361.6 ± 7.6	353.8 ± 7.6	332.7 ± 7.6	319.4 ± 7.7	*p* < 0.0001
Age (year)									
18–39	403.7 ± 7.0	382.6 ± 7.0	371.8 ± 7.0	367.9 ± 6.9	367.2 ± 7.1	353.3 ± 7.1	332.6 ± 7.2	328.2 ± 7.4	*p* < 0.0001
40–60	386.9 ± 11.6	360.5 ± 11.7	359.4 ± 11.7	356.5 ± 11.6	355.0 ± 11.7	353.4 ± 11.6	332.6 ± 11.6	314.9 ± 11.6	*p* < 0.0001
Sex									
Male	428.5 ± 8.2	394.8 ± 8.2	392.0 ± 8.2	387.2 ± 8.3	384.2 ± 8.3	379.5 ± 8.3	361.7 ± 8.3	353.4 ± 8.4	*p* < 0.0001
Female	362.9 ± 7.8	361.4 ± 7.8	348.9 ± 7.8	334.8 ± 7.8	333.3 ± 7.8	323.0 ± 7.8	306.1 ± 7.8	288.5 ± 7.9	*p* < 0.0001
Region									
North	397.6 ± 5.5	375.5 ± 4.7	370.4 ± 4.6	335.3 ± 4.2	330.8 ± 4.2	330.6 ± 4.2	320.0 ± 4.3	304.4 ± 4.5	*p* < 0.0001
Central	403.5 ± 4.8	392.8 ± 5.6	384.7 ± 5.8	382.8 ± 5.7	382.2 ± 5.7	375.5 ± 5.6	375.0 ± 5.8	356.2 ± 6.0	*p* < 0.0001
South	395.5 ± 14.9	363.0 ± 15.0	354.5 ± 14.9	346.4 ± 15.0	345.9 ± 14.9	339.1 ± 15.0	308.7 ± 15.0	304.8 ± 15.0	*p* < 0.0001
Income									
Low	431.6 ± 10.4	392.7 ± 10.5	390.6 ± 10.4	384.4 ± 10.3	381.6 ± 10.4	381.0 ± 10.4	343.3 ± 10.5	327.7 ± 10.6	*p* < 0.0001
Medium	404.4 ± 10.1	381.8 ± 10.1	371.3 ± 10.1	367.0 ± 10.1	366.7 ± 10.0	363.9 ± 10.1	334.6 ± 10.1	323.2 ± 10.2	*p* < 0.0001
High	365.5 ± 6.6	358.1 ± 6.7	349.7 ± 6.6	343.9 ± 6.7	343.6 ± 6.6	323.2 ± 6.6	318.4 ± 6.6	301.7 ± 6.7	*p* < 0.0001
Urbanicity									
Low	445.6 ± 18.0	409.5 ± 18.0	404.5 ± 18.0	388.7 ± 18.0	384.7 ± 18.0	378.3 ± 18.0	341.7 ± 18.0	329.4 ± 18.1	*p* < 0.0001
Medium	416.6 ± 11.7	404.0 ± 11.7	380.4 ± 11.7	377.7 ± 11.7	374.4 ± 11.6	360.1 ± 11.7	336.3 ± 11.7	297.8 ± 11.8	*p* < 0.0001
High	376.7 ± 10.2	368.1 ± 10.1	362.9 ± 10.2	332.0 ± 10.1	329.9 ± 10.1	305.0 ± 10.1	289.8 ± 10.1	286.3 ± 10.2	*p* < 0.0001

^1^ Mean ± SE (all such values). ^2^ Values adjusted for age, sex, region, income, and urbanicity.

**Table 4:** Daily fat intake (g), by age, sex, geographical region, income, and urbanicity, in Chinese rural adults from 1991 to 2011.

General Characteristics	Survey Year ^1^								*p*_trend_^2^
	1991	1993	1997	2000	2004	2006	2009	2011	
All	65.8 ± 4.1	68.1 ± 4.1	69.1 ± 4.1	70.8 ± 4.1	72.9 ± 4.1	73.8 ± 4.1	74.8 ± 4.1	76.9 ± 4.1	*p* < 0.0001
Age (year)									
18–39	63.6 ± 4.0	67.1 ± 4.2	68.8 ± 4.3	70.5 ± 4.2	70.9 ± 4.2	71.1 ± 4.2	73.6 ± 4.2	74.0 ± 7.2	*p* < 0.0001
40–60	66.4 ± 4.2	68.7 ± 4.1	69.3 ± 4.0	71.3 ± 4.0	75.1 ± 4.0	75.2 ± 4.0	76.1 ± 4.0	79.2 ± 4.0	*p* < 0.0001
Sex									
Male	71.3 ± 4.2	74.6 ± 4.2	75.5 ± 4.2	78.0 ± 4.2	80.3 ± 4.2	80.6 ± 4.2	81.3 ± 4.2	84.4 ± 4.2	*p* < 0.0001
Female	60.4 ± 3.8	62.0 ± 3.8	62.9 ± 3.8	63.9 ± 3.8	65.9 ± 3.8	67.4 ± 3.8	68.6 ± 3.8	69.8 ± 3.8	*p* < 0.0001
Region									
North	62.5 ± 2.7	65.3 ± 2.7	65.6 ± 2.7	66.9 ± 2.7	67.0 ± 2.7	68.3 ± 2.6	69.3 ± 2.6	70.4 ± 2.6	*p* < 0.0001
Central	57.8 ± 5.2	57.9 ± 5.2	61.7 ± 5.2	69.7 ± 5.1	71.5 ± 5.2	72.2 ± 5.2	72.8 ± 5.2	73.0 ± 5.2	*p* < 0.0001
South	75.2 ± 4.4	77.6 ± 4.4	78.5 ± 4.3	79.6 ± 4.4	79.8 ± 4.4	83.2 ± 4.4	83.4 ± 4.3	88.6 ± 4.4	*p* < 0.0001
Income									
Low	56.2 ± 3.1	58.5 ± 3.1	62.3 ± 3.1	63.5 ± 3.1	68.5 ± 3.1	68.8 ± 3.1	70.4 ± 3.1	71.3 ± 3.1	*p* < 0.0001
Medium	64.6 ± 4.5	67.2 ± 4.5	68.4 ± 4.5	69.5 ± 4.5	72.9 ± 4.5	75.4 ± 4.5	75.5 ± 4.5	76.7 ± 4.5	*p* < 0.0001
High	72.5 ± 4.2	73.4 ± 4.2	76.9 ± 4.1	77.4 ± 4.1	77.7 ± 4.1	79.4 ± 4.1	81.9 ± 4.1	83.9 ± 4.1	*p* < 0.0001
Urbanicity									
Low	55.3 ± 5.0	59.6 ± 5.0	59.7 ± 5.0	62.5 ± 5.0	62.9 ± 5.0	64.7 ± 5.0	66.1 ± 5.0	67.3 ± 5.0	*p* < 0.0001
Medium	59.9 ± 3.2	64.3 ± 3.2	65.0 ± 3.2	67.2 ± 3.2	74.1 ± 3.2	75.1 ± 3.1	77.4 ± 3.2	80.2 ± 3.2	*p* < 0.0001
High	71.0 ± 3.9	73.1 ± 3.9	76.6 ± 3.9	80.4 ± 3.9	81.7 ± 3.9	82.3 ± 3.9	84.7 ± 3.9	87.4 ± 3.9	*p* < 0.0001

^1^ Mean ± SE (all such values). ^2^ Values adjusted for age, sex, region, income, and urbanicity.

**Table 5:** Proportions of Chinese rural adults having more than 30% energy from fat from 1991 to 2011.

General Characteristics	Survey Year ^1^								*p*_trend_^2^
	1991	1993	1997	2000	2004	2006	2009	2011	
All	24.4 ± 0.6	25.4 ± 0.6	26.5 ± 0.6	34.8 ± 0.7	38.7 ± 0.6	47.9 ± 0.7	52.7 ± 0.7	58.3 ± 0.7	*p* < 0.0001
Age (year)									
18–39	24.9 ± 0.8	25.8 ± 0.8	26.2 ± 0.8	37.7 ± 0.9	33.1 ± 1.0	48.3 ± 1.1	51.6 ± 1.1	57.9 ± 1.3	*p* < 0.0001
40–60	23.6 ± 0.9	24.7 ± 1.0	27.8 ± 0.9	39.8 ± 0.9	35.9 ± 0.9	47.7 ± 0.9	53.4 ± 0.9	58.5 ± 0.9	*p* < 0.0001
Sex									
Male	26.0 ± 0.9	27.1 ± 0.8	27.6 ± 0.9	35.3 ± 0.9	39.0 ± 0.9	48.4 ± 0.9	53.2 ± 1.0	57.9 ± 1.1	*p* < 0.0001
Female	22.8 ± 0.8	23.4 ± 0.8	25.8 ± 0.8	34.3 ± 0.9	38.5 ± 0.9	47.5 ± 1.0	52.3 ± 1.0	58.6 ± 1.0	*p* < 0.0001
Region									
North	17.2 ± 1.1	19.4 ± 1.1	24.5 ± 1.2	35.9 ± 1.1	41.2 ± 1.2	44.9 ± 1.2	50.1 ± 1.2	55.1 ± 1.3	*p* < 0.0001
Central	16.7 ± 0.9	18.8 ± 1.0	27.4 ± 1.2	28.3 ± 1.0	36.9 ± 1.2	42.8 ± 1.2	46.1 ± 1.2	54.2 ± 1.3	*p* < 0.0001
South	29.0 ± 1.0	31.7 ± 1.0	35.3 ± 1.1	36.3 ± 1.0	43.2 ± 1.1	56.0 ± 1.2	61.8 ± 1.2	65.1 ± 1.2	*p* < 0.0001
Income									
Low	15.0 ± 0.9	15.5 ± 0.9	19.0 ± 0.9	22.7 ± 1.0	27.7 ± 1.0	36.9 ± 1.3	45.3 ± 1.3	51.7 ± 1.4	*p* < 0.0001
Medium	23.4 ± 1.0	23.6 ± 1.0	24.5 ± 1.1	33.0 ± 1.2	35.6 ± 1.1	44.7 ± 1.2	51.7 ± 1.2	58.4 ± 1.3	*p* < 0.0001
High	31.6 ± 1.1	32.1 ± 1.1	38.3 ± 1.1	46.7 ± 1.1	51.4 ± 1.1	59.1 ± 1.1	59.3 ± 1.1	62.8 ± 1.2	*p* < 0.0001
Urbanicity									
Low	11.2 ± 0.8	14.3 ± 0.9	18.1 ± 0.9	19.1 ± 0.9	22.2 ± 1.0	30.6 ± 1.1	36.0 ± 1.2	45.2 ± 1.3	*p* < 0.0001
Medium	18.7 ± 1.0	21.5 ± 1.0	23.5 ± 1.0	33.0 ± 1.1	38.5 ± 1.1	47.9 ± 1.2	53.2 ± 1.2	56.9 ± 1.3	*p* < 0.0001
High	34.6 ± 1.1	35.9 ± 1.1	43.7 ± 1.1	51.9 ± 1.2	57.0 ± 1.1	62.9 ± 1.1	67.9 ± 1.1	70.8 ± 1.1	*p* < 0.0001

^1^ Mean ± SE (all such values). ^2^ Values adjusted for age, sex, region, income, and urbanicity.

**Table 6:** Daily protein intake (g), by age, sex, geographical region, income, and urbanicity, in Chinese rural adults from 1991 to 2011.

General Characteristics	Survey Year ^1^								*p*_trend_^2^
	1991	1993	1997	2000	2004	2006	2009	2011	
All	75.3 ± 1.5	74.7 ± 1.5	73.9 ± 1.5	71.4 ± 1.5	70.2 ± 1.5	69.6 ± 1.5	69.3 ± 1.5	64.4 ± 1.5	*p* < 0.0001
Age (year)									
18–39	76.9 ± 1.4	75.9 ± 1.4	74.8 ± 1.4	70.6 ± 1.4	69.3 ± 1.4	68.9 ± 1.4	68.7 ± 1.4	65.0 ± 1.5	*p* < 0.0001
40–60	73.3 ± 1.9	73.4 ± 1.9	73.1 ± 1.9	72.7 ± 1.9	71.0 ± 1.9	70.8 ± 1.9	70.0 ± 1.9	64.1 ± 1.9	*p* < 0.0001
Sex									
Male	80.6 ± 1.8	79.7 ± 1.8	79.3 ± 1.8	78.0 ± 1.8	76.3 ± 1.8	75.5 ± 1.8	75.2 ± 1.8	71.3 ± 1.8	*p* < 0.0001
Female	71.0 ± 1.4	70.4 ± 1.4	67.5 ± 1.4	65.0 ± 1.4	64.4 ± 1.3	64.3 ± 1.2	63.5 ± 1.4	58.1 ± 1.4	*p* < 0.0001
Region									
North	78.1 ± 2.5	77.5 ± 2.5	72.6 ± 2.5	71.4 ± 2.5	66.9 ± 2.5	66.4 ± 2.4	64.9 ± 2.5	63.5 ± 2.5	*p* < 0.0001
Central	75.7 ± 3.7	74.9 ± 3.7	75.5 ± 3.7	73.5 ± 3.7	72.5 ± 3.7	71.8 ± 3.7	70.7 ± 3.7	69.8 ± 3.7	*p* < 0.0001
South	72.8 ± 2.4	72.4 ± 2.4	72.5 ± 2.4	71.5 ± 2.4	70.7 ± 2.4	68.0 ± 2.4	66.7 ± 2.4	61.6 ± 2.4	*p* < 0.0001
Income									
Low	74.2 ± 2.1	72.7 ± 2.1	72.1 ± 2.1	70.3 ± 2.1	68.7 ± 2.1	68.4 ± 2.1	67.1 ± 2.1	62.3 ± 2.1	*p* < 0.0001
Medium	75.8 ± 1.4	74.6 ± 1.4	74.0 ± 1.4	71.6 ± 1.4	69.8 ± 1.4	69.6 ± 1.4	69.0 ± 1.4	66.2 ± 1.4	*p* < 0.0001
High	76.3 ± 1.5	76.2 ± 1.5	73.4 ± 1.5	72.4 ± 1.5	72.3 ± 1.5	71.0 ± 1.5	71.9 ± 1.5	66.8 ± 1.5	*p* < 0.0001
Urbanicity									
Low	74.7 ± 2.3	74.0 ± 2.3	71.1 ± 2.3	70.8 ± 2.3	68.6 ± 2.3	68.6 ± 2.3	66.5 ± 2.3	65.2 ± 2.3	*p* < 0.0001
Medium	73.7 ± 1.6	73.3 ± 1.6	72.5 ± 1.6	71.2 ± 1.6	71.5 ± 1.6	69.7 ± 1.6	70.1 ± 1.6	61.9 ± 1.6	*p* < 0.0001
High	77.6 ± 1.5	77.0 ± 1.5	75.3 ± 1.5	73.0 ± 1.5	72.3 ± 1.5	71.2 ± 1.5	71.4 ± 1.5	66.7 ± 1.5	*p* < 0.0001

^1^ Mean ± SE (all such values). ^2^ Values adjusted for age, sex, region, income, and urbanicity.
